# Air ions and mood outcomes: a review and meta-analysis

**DOI:** 10.1186/1471-244X-13-29

**Published:** 2013-01-15

**Authors:** Vanessa Perez, Dominik D Alexander, William H Bailey

**Affiliations:** 1 Exponent, Inc., Health Sciences, Center for Epidemiology, Biostatistics, and Computational Biology, 525 West Monroe Street, Suite 1050, 60661, Chicago, IL, USA; 2 Exponent, Inc., Health Sciences, Center for Epidemiology, Biostatistics, and Computational Biology, 4141 Arapahoe Avenue, Suite 101, 80303, Boulder, CO, USA; 3 Exponent, Inc., Health Sciences, Center for Exposure Assessment and Dose Reconstruction, 17000 Science Drive, Suite 200, 20715, Bowie, MD, USA

**Keywords:** Mood disorders, Depression, Air ionization, Ion exposure, Epidemiology, Systematic review, Negative ion, Positive ion

## Abstract

**Background:**

Psychological effects of air ions have been reported for more than 80 years in the media and scientific literature. This study summarizes a qualitative literature review and quantitative meta-analysis, where applicable, that examines the potential effects of exposure to negative and positive air ions on psychological measures of mood and emotional state.

**Methods:**

A structured literature review was conducted to identify human experimental studies published through August, 2012. Thirty-three studies (1957–2012) evaluating the effects of air ionization on depression, anxiety, mood states, and subjective feelings of mental well-being in humans were included. Five studies on negative ionization and depression (measured using a structured interview guide) were evaluated by level of exposure intensity (high vs. low) using meta-analysis.

**Results:**

Consistent ionization effects were not observed for anxiety, mood, relaxation/sleep, and personal comfort. In contrast, meta-analysis results showed that negative ionization, overall, was significantly associated with lower depression ratings, with a stronger association observed at high levels of negative ion exposure (mean summary effect and 95% confidence interval (CI) following high- and low-density exposure: 14.28 (95% CI: 12.93-15.62) and 7.23 (95% CI: 2.62-11.83), respectively). The response to high-density ionization was observed in patients with seasonal or chronic depression, but an effect of low-density ionization was observed only in patients with seasonal depression. However, no relationship between the duration or frequency of ionization treatment on depression ratings was evident.

**Conclusions:**

No consistent influence of positive or negative air ionization on anxiety, mood, relaxation, sleep, and personal comfort measures was observed. Negative air ionization was associated with lower depression scores particularly at the highest exposure level. Future research is needed to evaluate the biological plausibility of this association.

## Background

Several experimental human studies on air ion exposure and mood ratings have been published throughout the years. While their evidence is inconsistent, the findings have increased awareness of mood alterations possibly associated with such exposure. Ions are ubiquitous, whereby any molecule with an unbalanced electron to proton ratio results in a net positive or negative electrical charge [1]. Air ions are produced from alterations in the atmosphere and weather phenomena, by natural radioactivity, and by combustion processes [2,3]. They are also generated by air ionizers sold commercially and by corona activity on the surface of high voltage conductors of transmission lines.

Some experimental research indicates that exposure to negative air ions is linked to reduced depression severity [4-8], lower psychological stress [9], less anxiety [10], and enhanced well-being [11-14]. Others suggest that exposure to positive air ions may be associated with feelings of unpleasantness, irritability, and heightened anxiety [15-17]; while some have found no mood alterations associated with air ionization [18,19].

Historically, evidence of psychological measures and air ionization has been equivocal because research findings use heterogeneous experimental protocols evaluating diverse study populations; use various methods to measure mood-related outcomes; and use inadequate experimental design and procedures including control over relevant exposures [20]. Diagnostic trends for classifying mood disorders and technological advancements in environmental therapies (e.g. air ionization systems) have likely influenced study findings. Furthermore, most studies have tested relatively small study populations. To the best of our knowledge, no current review has summarized the possible effects on mood and well-being attributed to air ionization. We therefore conducted a structured literature review to evaluate human experimental studies on positive and negative air ion exposure and ratings of depression, anxiety, mood states, and subjective feelings of mental well-being. In addition, we quantitatively examined negative air ionization and depression symptom severity using meta-analysis.

## Methods

### Literature search and study identification

A structured literature review performed for the Minnesota Environmental Quality Board on the biological/health effects attributed to air ions and direct current transmission lines was used to identify the historical literature up to 1982 [21]. We further conducted a structured literature search using Medline (PubMed) to identify experimental studies published between 1 January 1982 and August, 2012 on air ionization and depression, anxiety, mood states, and subjective feelings of mental well-being in humans. ProQuest DIALOG was used to retrieve studies from the environmental and behavioral sciences, engineering, and other technical databases, including Elsevier, Biobase, and Embase.

Identical search strings for PubMed and ProQuest DIALOG referenced the exposure (air ions, charged aerosols, corona ions, atmospheric ions, ionization, ionized air, heavy ions, light ions) and outcomes of interest (depression, anxiety, mood, activation, personal comfort, relaxation, sleepiness). We manually reviewed reference lists in all retrieved articles for related publications. Thirty-three English-language studies published between 1957 and 2012 met our inclusion criteria (Table 1).

**Table 1:** Study characteristics

**Author and year**	**Study objective**	**Study design**	**Blinding**	**Study population**	**Total sample size**
Silverman and Kornblueh 1957[27]^a,b^	Evaluate effect of negative and positive ions on the human electroencephalogram and sleep	Crossover experiment	Not reported	10 healthy adults and 2 additional subjects with chronic stationary neurologic conditions	12
McGurk, 1959[17]^c^	Evaluate effect of negative and positive ions on self-reported feelings of comfort	Crossover experiment	Single-blind (subjects)	10 college-aged males	10
Yaglou, 1961[19]^b^	Evaluate effect of negative and positive ions on relaxation	Crossover experiment	Single-blind (subjects)	25 healthy adults (age range: 22–51)	25
Yaglou, 1961[19]^b^	Evaluate effect of negative and positive ions on relaxation and sleepiness	Crossover experiment	Single-blind (subjects)	6 arthritic patients (age range: 30–62)	6
Assael et al., 1974[11]^b^	Evaluate effect of negative ions on human electroencephalogram	Crossover experiment	Double-blind	10 healthy participants (age range: 20–65) and 10 subjects receiving tranquilizers	20
Albrechtsen et al., 1978[37]^b,c^	Evaluate effect of negative and positive ions on human well-being and mental performance	Crossover experiment	Single-blind (subjects)	Study 1: six women (age range: 20–30) chosen at random; study 2: 12 subjects (age range: 19–45) selected because they appeared to be most sensitive to ionization	Study 1: 6 Study 2: 12
Charry and Hawkinshire, 1981[15]^a^	Evaluate effect of negative and positive ions on mood	Crossover experiment	Single-blind (subjects)	85 adults (age range: 18–60; mean age: 30)	85
Hawkins, 1981[38]^b, c^	Evaluate effect of negative and positive ions on subjective well-being and comfort	Crossover experiment	Double-blind	Study groups based on three areas of variable air ionization levels within the building (area 1: 20 women; area 2: 32 adults; and area 3: 54 adults)	Area 1: 20
					Area 2: 22
					Area 3: 54
Tom et al., 1981[34]^a,b^	Evaluate effect of negative ions on human performance and mood	Randomized controlled trial	Double-blind	56 adults (age range: 17–61; mean age: 23)	56
Buckalew and Rizzuto, 1982[12]^a,b^	Evaluate effect of negative ions on subjective feelings of mood and psychological state	Randomized controlled trial	Double-blind	Two groups of 12 paid male volunteers matched on age, education, physical condition, and smoking habits (age range: 20–30; mean age: 22.8)	24
Dantzler et al., 1983[25]^a^	Evaluate effect of positive and negative ions on somatic symptoms and mood changes	Crossover experiment	Double-blind	9 patients with bronchial asthma (age range: 35–64)	9
Baron et al., 1985[28]^a^	Evaluate effect of negative ions on self-reported affect/mood	Crossover experiment	Single-blind (subjects)	71 male undergraduate students	71
Deleanu and Stamatiu, 1985[29]^a,b,d^	Evaluate effect of negative ions on psychiatric symptoms	Experimental (no control group)	Not reported	112 patients with neurasthenias, psychoses, or personality disorders	112
Gianinni et al., 1986[16]^a^	Evaluate effect of negative and positive ions on anxiety, excitement, and suspicion	Crossover experiment	Double-blind	14 university-affiliated volunteers	14
Gianinni et al., 1986/1987[30]^a^	Evaluate effect of positive ions on anxiety and excitement	Crossover experiment	Double-blind	12 adult male volunteers	12
Finnegan et al., 1987[40]^c^	Evaluate effect of negative ions on personal comfort rating	Crossover experiment	Single-blind (subjects)	26 adults working within 5 different rooms of an office building	26
Hedge and Collis, 1987[18]^a^	Evaluate effect of negative ions on mood	Crossover experiment	Double-blind	28 healthy women (age range: 19–58)	28
Lips et al., 1987[13]^b,c^	Evaluate effect of negative ions on well-being and comfort	Crossover experiment	Double-blind	18 normal, healthy employees working in one of two rooms, whereby room 1 had windows providing air ventilation and room 2 was mechanically ventilated	18
Misiaszek et al., 1987[14]^a,b^	Evaluate effect of negative ions on manic behavior and sleep	Experimental (phase I: no control group; phase II: with-in subjects, repeated measures)	Phase I: No Blinding Phase II: Double-blind	8 manic patients (age range: 22–49)	Phase I: 4 Phase II: 4
Reilly and Stevenson, 1993[33]^a^	Evaluate effect of negative ions on anxiety	Crossover experiment	Single-blind (subjects)	8 healthy men (age range: 19–25)	8
Terman and Terman, 1995[6]^d^	Evaluate effect of negative ions on seasonal depression	Randomized controlled trial	Double-blind	25 patients (mean age: 38.2 ± 11) with winter depression	Low-density negative ion group: 13 High-density negative ion group: 12
Watanabe et al., 1997[35]^a,c^	Evaluate effect of negative ions on mood and pleasantness	Crossover experiment	Single-blind (subjects)	13 healthy adults (age range: 21–49; mean age: 26.4)	13
Terman et al., 1998[8]^b,d^	Evaluate effect of negative ions on sleep and seasonal depression	Crossover experiment	Double-blind	124 subjects (age range: 18–59; mean age: 39.4 ± 9.8) with seasonal affective disorder	124 (20 randomized to high-density negative ionization and 19 randomized to low-density negative ionization)
Nakane et al., 2002[10]^a^	Evaluate effect of negative ions on anxiety	Crossover experiment	Not reported	12 female undergraduates (age range: 18–22)	12
Iwama et al., 2004[39]^b^	Evaluate effect of negative ions on tension	Randomized controlled trial	Double-blind	44 patients randomized to the control and 51 patients randomized to receive treatment (mean age among men: 37 ± 18; mean age among women: 43 ± 20)	95
Goel et al., 2005[22]^b,d^	Evaluate effect of negative ions on sleep and chronic depression	Randomized controlled trial	Double-blind	32 patients (age range: 22–65; mean age: 43.7 ± 12.4) with non-seasonal chronic depression	32 (22 randomized to low- or high-density)
Goel and Etwaroo, 2006[5]^a,b,d^	Evaluate effect of negative ions on depression, total mood disturbance, and anger	Randomized controlled trial	Single-blind (subjects)	118 mildly depressed and non-depressed college students (mean age: 19.4 ± 1.7)	118 (59 randomized to low or high density)
Terman and Terman, 2006[7]^b,d^	Evaluate effect of negative ions on sleep and seasonal depression	Randomized controlled trial	Double-blind	99 adults with seasonal depression (94 cases) and bipolar II disorder (five cases) (age range: 19–63; mean age: 40.4 ± 10.4)	99 (39 randomized to low or high density)
Gianinni et al., 2007[26]^a^	Evaluate effect of negative ions on manic states	Crossover experiment	Double-blind	24 manic male patients (age range: 23–29; mean age: 26.7)	24 (20 analyzed)
Malcolm et al., 2009[32]^a,b^	Evaluate effect of negative ions on positive affective memory	Randomized controlled trial	Single-blind (subjects)	30 healthy subjects (age range: 18–28) randomized to either receive high-density negative air ion exposure or to a control condition	30
Flory et al., 2010[4]^d^	Evaluate effect of negative ions on seasonal depression	Randomized controlled trial	Single-blind (subjects)	73 university-affiliated women (age range: 18–51; mean age: 20.8 ± 5.69) with seasonal affective disorder	73 (38 randomized to low or high density)
Malik et al., 2010[9]^a^	Evaluate effect of negative ions on psychological stress	Crossover experiment	Single-blind (subjects)	20 regular users of computers as part of their job (age range: 24–35; mean age: 28.9)	20
Dauphinais et al., 2012[24]^d^	Evaluate the effect of negative air ions on seasonal depression	Randomized controlled trial	Double-blind	44 adult patients (20 in the low-density group) with bipolar depression	20
Harmer et al., 2012[31]^a,b,d^	Evaluate the effect of high-density negative air ions on emotional processing in patients with seasonal depression	Randomized controlled trial	Double-blind	21 adult patients with seasonal depression; 21 controls. Mean ages of groups between 30–35 years	42

^a^Activation, anxiety, mood.

^b^Relaxation and sleep.

^c^Personal comfort rating.

^d^Depression.

Inclusion criteria consisted of experiments among subjects exposed to negatively- or positively-charged small air ions, or both; studies published in the English language; and studies that reported associations between ionization and mood indicators (e.g., depression, anxiety, mood states, and reports of mental well-being). No restrictions on the number of subjects evaluated in each study were required. Animal studies, letters to the editor and editorials, references not reporting original data, and studies with no relevant exposure or outcome were excluded.

### Data extraction and statistical methods

Qualitative information (study population/design, ion polarity/concentration, exposure duration) and quantitative data (mood indicator effects) were extracted. Studies were consolidated qualitatively into four outcome categories: activation, anxiety, and mood; relaxation/sleep; personal comfort ratings; and depression.

A meta-analysis was performed on five studies [4,6-8,22] of negative air ionization and depression symptom severity as measured using the 29-item Structured Interview Guide for the Hamilton Depression Rating Scale, Seasonal Affective Disorders (SIGH-SAD), which consists of the 21-item Hamilton Depression Rating Scale and the 8-item Atypical Scale. Forest plots from random effects modeling [23] were generated to estimate weighted group mean differences in depression scores, 95% confidence intervals (CIs), and corresponding *p-*values for heterogeneity. Of note, using the random effects analysis, the weighted mean is defined as the sum of each study effect size multiplied by its weight (i.e., the inverse of the within-study variance plus the between-studies variance) divided by the sum of the weights. The variance of the weighted group mean difference is defined as the reciprocal of the sum of the weights. The data from Terman and Terman [6] were extracted from their Figure one; exact values have been requested from the authors. Depression score data before and after exposure to low density ions were also requested from Dauphinais et al. [24] for possible inclusion. Sensitivity analyses were performed to examine data robustness. Publication bias was assessed using funnel plots, the Begg rank correlation test, and Egger’s regression analysis. All analyses were performed using the Comprehensive Meta-Analysis software (version 2.2.046; Biostat, Englewood, NJ). Additional dose–response relationships were evaluated by plotting exposure duration by depression score mean differences and their corresponding 95% CIs in Microsoft Excel (2010).

## Results

Studies meeting inclusion criteria are summarized in Table 1. All studies included adults only and sample sizes ranged from 4 to 124 participants. Apart from the studies that evaluated ion effects on patients with some form of depression, six studies [11,14,19,25-27] also evaluated the influence of ions on mood states of persons with varying health conditions. Collectively, the findings from these six studies did not provide contrasting results from those studies that included only healthy subjects. Most studies examined negative air ionization only (n=24); one examined positive air ionization only; and eight studied the effects of both. Blinding of study subjects was not reported in three experiments, nor was it obvious upon review of the study methodology. Among the 30 studies that conducted blind experiments, 18 were double-blind. All but one study [19] was published in a peer-reviewed journal.

Air ion intensities and duration are summarized in Table 2. Air ion intensities were reported in 29 studies (range: 1000 ions/cm^3^ (ambient levels) to 27,500,000 ions/cm^3^). Air ionization duration ranged from 10 minutes at a single time point, to daily treatment periods administered for multiple days, to successive weeks at a time where air ion generators were switched on continuously. Collectively, many studies reported a mood-related response after exposure to ionized air; however, considerable variation by outcome, statistical significance testing, and degree of precision across the reported data was noted.

**Table 2:** Air ion exposure assessment, psychological measures, and study findings

**Author and year**	**Air ion exposure (Duration)**	**Ion concentration**	**Metrics used for mental health outcomes**	**Primary findings**
Silverman and Kornblueh 1957[27]^a,b^	Negative air ion (30 minutes)	Not reported	Human electroencephalogram	Decrease in alpha frequency in most subjects; half of the subjects reported relaxation and sleepiness with ionization (slightly more frequent for (−) than (+) ion exposure); one consistent finding was a decrease in alpha frequency during negative or positive ionization (or both) in all but two subjects. Findings reported as “transient.”
	Positive air ion (30 minutes)		Activations by hyperventilation, apnea, photic stimulation and sleep (natural)	
McGurk, 1959[17]^c^	Negative air ion (5 hours)	8.0 × 10^3^ ions/cm^3^	Self-reported feelings of comfort, ease by which subjects worked on a cognitive task, and reactions to the test room atmosphere	Regarding negative ionization, a significant percent of subjects appeared to detect ionization condition despite blinding and reported more pleasant feelings.
	Positive air ion (2 hours)			Regarding positive ionization, subjects reported more unpleasant feelings.
Yaglou, 1961[19]^b^	Negative air ion (1–2 hours)	5,000 to 10,000 ions/cm^3^ air	Self-reported impressions (indifference, relaxation, air freshness, headache, respiratory irritation, restlessness)	Subjectively, positive air ions seemed to increase the incidence of upper respiratory irritation in the winter, while negative air ions had little or no effect on the quality of air.
	Positive air ion (1–2 hours)			5% of subjects reported feeling relaxed when exposed to positive air ions; 17% reported feeling relaxed when exposed to negative air ions; and 21% of subjects reported feeling relaxed under control conditions
Yaglou, 1961[19]^b^	Negative air ion (1–2 hours)	10^5^-10^6^ ions/cm^3^ air	Self-reported impressions (indifference, relaxation, air freshness, headache, respiratory irritation, restlessness)	Subjectively, negative air ions did not alleviate joint symptoms, while positive air ions seemed to make the symptoms worse; a higher frequency of patients reported feeling relaxed or sleepy, or both when exposed to negative versus positive air ions
	Positive air ion (1–2 hours)			
Assael et al., 1974[11]^b^	Negative air ion (45 minutes)	3.5 × 10^5^ ions/cm^3^	EEG parameters:	Decrease in alpha frequency manifestation of general relaxation induced by negative air ions. Increase of amplitude interpreted as improvement of perception and apperception. Subjectively, all patients experienced initial relaxation followed by alertness connected with moving of alpha-waves from occipital to frontal areas.
			frequency (Hz)	
			amplitude (μV)	
			spreading of alpha waves area	
			synchronization of right and left hemispheres	
			Self-reported relaxation, alertness, working capacity, relief	
Albrechtsen et al., 1978[37]^b,c^	Negative air ion (Experiment I: 8 hours; Experiment II: 15 minutes) Positive air ion (Experiment I: 8 hours; Experiment II: 15 minutes)	300-9,000 ions/cm^3^	Mental performance:	No significant effects of positive or negative air ions found.
			number of tasks per hour	
			Subjective voting based on % scale:	
			extent of exertion	
			perception on air quality	
			perception of tasks	
			current feeling (sleepy vs. alert)	
Charry and Hawkinshire, 1981[15]^a^	Positive air ion (1.5 hours )	Positive air ions: 2.0-3.0 × 10^4^ ions/cm^3^	Mood Adjective Check List	For most subjects, mood changes induced by air ion exposure characterized by increased tension and irritability.
	Ambient condition (contained both) (1.5 hours)	Ambient: 3.0 × 10^2^ ions/cm^3^	Sharav Questionnaire (mood)	
Hawkins, 1981[38]^b, c^	Negative air ion (Weeks 5 to 12- on continuously)	Negative air ion: 2.0-3.5 × 10^3^ ions/cm^3^	Personal ratings of thermal comfort, stuffiness, alertness, well-being	Negative air ion exposure associated with higher subjective ratings of alertness, atmospheric freshness, environmental/personal warmth, and a reduction in the overall complaint rate by 50%. Night-shift working was associated with discomfort and ill-health. Positive air ion effects were not explicitly discussed.
	Positive air ion (Weeks 5 to 12- on continuously)	Positive air ion: 50–125 ions/cm^3^ air		
Tom et al., 1981[34]^a,b^	Negative air ion (15 minutes)	Negative air ion: 16,160 ions/cm^3^	Likert scale survey for psychological state (difficulty of concentration, energetic, mood state, sociable, relaxed)	Subjects reported being more energetic and finding it easier to concentrate under the experimental condition than the control condition. Negative air ion exposure had a positive effect on certain aspects of human performance and mood.
		Control (natural environment): 204.4 ions/cm^3^		
Buckalew and Rizzuto, 1982[12]^a,b^	Negative air ion (6 hours)	Not reported	Taylor Manifest Anxiety Scale (TMAS)	Mood index data showed significant changes in the subjective perception of both physiological state (relaxation increased) and psychological state (irritability, depression, and tenseness decreased while calmness and stimulation increased).
			Self-report Mood Index	
Dantzler et al., 1983[25]^a^	Negative air ion (6 hours)	Negative and positive air ions: 60,000-110,000 ions/cm^3^	Sharav Questionnaires 1 and 2 Mood Adjective Check List	Patients’ mood did not differ significantly between the two ion exposures.
	Positive air ion (6 hours)			
Baron et al., 1985[28]^a^	Negative air ion (20 minutes)	Ambient condition: 2.0-3.0 × 10^2^ ions/cm^3^	Self-reported affect (Profile of Mood States survey)	Exposure to moderate or high levels of negative air ions significantly enhanced aggression by Type A subjects, but not among others. Negative air ions produced positive shifts in reported moods in the absence of provocation, but negative shifts in moods in the presence of provocation.
		Moderate condition: 4 × 10^4^ ions/cm^3^	Aggression measured by mean level of heat selected by subjects on each of the 20 occasions when the red light appeared	
		High condition: 7.0-8.0 × 10^4^ ions/cm^3^	Memory measured by the number of traits and the number of behaviors subjects could recall about the accomplice	
Deleanu and Stamatiu, 1985[29]^a,b,d^	Negative aeroionotherapy (daily treatment of 15–50 minutes for 10–30 days)	1-1.5 × 10^4^ ions/cm^3^	Amelioration of asthenia, depressive reactions, anxiety, excitability and irascibility, cephalea, insomnia, and general disposition in patients with various psychiatric disorders	In most treated patients, a diminution or even the disappearance of the target symptoms was obtained (asthenia, depressive reactions, anxiety, irascibility, cephalea, insomnia, and general disposition).
Gianinni et al., 1986[16]^a^	Negative air ion (20 minutes)	Negative air ion: not reported	Brief Psychiatric Rating Scale	Cations were found to increase anxiety, excitement, and suspicion. Anions reversed the effects of cations and, in addition, reduced suspicion and excitement to levels below those occurring before cationization.
	Positive air ion (20 minutes)			
		Positive air ion: 2.9 × 10^3^ ions/cm^3^		
Gianinni et al., 1986/87[30]^a^	Positive air ion (2 hours)	2,050-2,300 ions/cm^3^	Brief Psychiatric Rating Scale	Symptoms of anxiety and excitement significantly increased. During the time of exposure, serum serotonin levels also increased significantly.
Finnegan et al., 1987[40]^c^	Negative air ion (6–8 weeks)	1.84 × 10^3^ ions/cm^3^	Personal comfort rating	No significant effect on personal comfort found. Effects on symptoms were non-significant except for URTI and nausea in the high negative air ion period.
Hedge and Collis, 1987[18]^a^	Negative air ion (7 hours)	2 × 10^4^ ions/cm^3^	Mood Adjective Check List	Evidence for beneficial effects of negative air ions on mood and performance could not be demonstrated.
			Two cognitive tasks:	
			naming 24 different colors printed on card	
			Stroop Colour Word test	
Lips et al., 1987[13]^b,c^	Negative air ion Weeks 2 and 4 - on continuously; Week 3 - mornings only	5 × 10^4^ ions/cm^3^	10 linear scales (rated 0 to 10) on which each subject was asked to assess his or her well-being and the quality of the environment	After their exposure to enhanced negative air-ion concentrations, the subjects' assessments of both their own well-being and the quality of the environment improved significantly: neither harmful effects of exposure to enhanced levels of negative air ions nor changes in perceived thermal comfort were detected.
Misiaszek et al., 1987[14]^a,b^	Negative air ion Phase I: 1 hour; Phase II: 1.5 hours	Phase I: 40,000-60,000 small, 50–1000 medium, 50–4,000 large ions/cm^3^	State Trait Anxiety Inventory and Inpatient Multidimensional Psychiatric Scale	Phase I: All subjects fell to sleep, reported being calm afterwards; manic behavior reappeared 5–10 minutes after treatment
				Phase II: 3/4 subjects fell to sleep, 1 subject appeared less agitated; manic behavior reappeared 5–10 minutes after treatment
		Phase II: 50,000-70,000 small, 50–3,200 medium, 50–7,000 large ions/cm^3^		
Reilly and Stevenson, 1993[33]^a^	Negative air ion (30 minutes pre-test + 40 minutes during test)	1.72 × 10^5^ ions/cm^3^	Measurements were made of state anxiety according to Spielberger et al. (1970)	There was no significant effect of air ions on state anxiety pre-or post-exercise or on the perception of effort.
Terman and Terman, 1995[6]^d^	Negative air ion (30 minute sessions for 20 days)	Low density: 1.0 × 10^4^ ions/cm^3^	SIGH – SAD	The severity of depressive symptoms decreased selectively for the group receiving high-density treatment. When a remission criterion of 50% or greater reduction in symptom frequency/severity was used, 58% of subjects responded to high-density treatment while 15% responded to low-density treatment.
			Clinical Global Impressions Scale	
		High density: 2.7 × 10^6^ ions/cm^3^		
Watanabe et al., 1997[35]^a,c^	Negative air ion (10 minutes)	2.0 × 10^4^ ions/cm^3^	Self-reported feelings of temperature, pleasantness, fatigue, and sweating	There were no differences in the moods of these persons or changes in their blood pressures between the two saunas.
Terman et al., 1998[8]^b,d^	Negative air ion (30 minutes per day for 10–14 days)	Low density: 1.0 × 10^4^ ions/cm^3^High density: 2.7 × 10^6^ ions/cm^3^	SIGH – SADSelf-rating version of the SIGH-SADSleep patterns	Improved depression rating of 42-50% and 20-40% remission rate. Described as a "small effect" in period 1 and "large effect" in period 2. Analysis of depression scale percentage change scores showed low-density air ion response to be inferior to all other groups, with no other group differences. Sleep measures subjects given morning light awakened 0.62 ± 0.62 hours earlier than at baseline; negative air ions, 0.41 ± 0.37 hours earlier; and evening light, 0.09 ± 0.58 hours earlier.
Nakane et al., 2002[10]^a^	Negative air ion (40 minutes during task or 30 minutes post-task)	5.5-7.3 × 10^3^ ions/cm^3^	Japanese version of the State-Trait Anxiety Inventory, Anxiety StateSalivary cortisol and chromogranin A-like immunoreactivityTask performance	The increase in the CgA-like IR level was attenuated by the exposure to negative air ions during the task. The exposure to air ions during the recovery period following the task was effective for rapidly decreasing the CgA-like IR level that had increased after the task. These effects by negative air ions were also observed using STAI-S. Task performance was slightly but significantly improved by the presence of negative air ions.
Iwama et al., 2004a[39]^b^	Negative ion (not reported)	3000 parts/cm^3f^	Degree of tension: 1 = relaxed; 2 = normal tension; 3 = mild tension; 4 = moderate tension; and 5 = severe tension	Degree of tension decreased significantly and more rapidly in the negative ion-rich environment.
Goel et al., 2005[22]^b,d^	Negative ion (1 hour upon wakening for 5 weeks)	Low density: 1.7 × 10^11^ ions/s [1 × 10^4^ ions/cm^3^]^e^	SIGH – SAD	SIGH-SAD score improvement was 51.1% for high-density ions v. 17.0% for low-density ions. Remission rates were 50% and 0%, respectively.
		High density: 4.5 × 10^14^ ions/s [2.7 × 10^7^ ions/cm^3^]^e^	Evening saliva samples obtained before and after treatment for ascertainment of circadian melatonin rhythm phase	
Goel and Etwaroo, 2006[5]^a,b,d^	Negative ion (30 minutes for three consecutive evenings)	Low density: 1.7 × 10^11^ ions/s [1 × 10^4^ ions/cm^3^]^e^High density: 4.5 × 10^14^ ions/s [2.7 × 10^7^ ions/cm^3^]^e^	BDI	The three active stimuli (bright light, auditory stimuli, or high-density negative ion exposure), but not the low-density placebo, reduced depression, total mood disturbance and/or anger within 15–30 min.
			The Profile of Mood	
			States Questionnaire	
			The Karolinska Sleepiness Scale	
			Likert scales assessed four aspects of stimulus perception using a 7-point scale. Subjects rated stimulus hedonics and intensity, as well as its effects on mood and on alertness	
Terman and Terman, 2006[7]^b,d^	Negative ion (93 minutes before waking up)	Low density: 1.7 × 10^11^ ions/s [1 x 10^4^ ions/cm^3^]^e^	SIGH – SAD	Post-treatment improvement results were high-density ions, 47.9%; and low-density ions, 22.7% (significantly different).
		High density: 4.5 × 10^14^ ions/s [2.7 x 10^7^ ions/cm^3^]^e^	Emergence or exacerbation of depression, sleep, appetite/weight, headache	
Gianinni et al., 2007[26]^a^	Negative ion (1 hour)	3 × 10^3^ ions/cm^3^	Brief Psychiatric Rating Scale	A significant anti-manic effect was observed: total rating scores declined with anion treatment.
Malcolm et al., 2009[32]^a,b^	Negative ion (30 minutes pre-test and 60 minutes during test)	Not reported	Subjective state measured by six visual analogue scales for happiness, sadness, hostility, alertness, anxiety and calmness.	Association between BDI score and treatment; increased recall and recognition of positive terms versus negative terms; findings indicate that HDNI treatment produces a positive bias in emotional recall and recognition.
			The emotional test battery consisted of an emotional categorization task with surprise emotional recall and recognition, a facial expression recognition test, and a dot-probe task of attention with masked and unmasked conditions.	
Flory et al., 2010[4]^d^	Negative ion (30 minutes for 12 days)	Low density: 4.0 × 10^3^ ions/cm^3^	SIGH – SAD–Self Rating:	Subjects in all four groups showed significant score decreases on the SIGH-SAD-SR and on the BDI. For raw scale scores, neither main effects of treatment nor interactions between treatment and time were significant. When remission outcome criteria were used, exposure to high-density negative ions was more effective than either of the two placebo conditions, although the difference was not significant.
		High density: ≥ 2.0 × 10^6^ ions/cm^3^	BDI	
			Diagnostic and Statistical Manual of Mental Disorders (DSM-IV) criteria for SAD	
Malik et al., 2010[9]^a^	Negative ion (2 hours)	>1,000,000 counts/cm^3^	Self-reported computer-oriented stress, physiological and psychological stress	A significant decline in computer-oriented stress and psychological stress was noticed post-computer operations in presence of negative ions.
Dauphinais et al., 2012[24]^d^	Negative ion (7.5 min/day or 15 min/day if tolerable for 8 weeks)	1.7 × 10^11^ ions/s [1 × 10^4^ ions/cm^3^]^e^	SIGH-SAD	No significant difference in SIGH-SAD scores between light therapy and low-density negative ion groups at study end or in the proportions of responders or remitters in these groups.
Harmer et al., 2012[31]^a,b,d^	Negative ion (30 minutes pre-test and 60 minutes during test)	Not reported	Subjective state measured by six visual analogue scales (happiness, surprise, sadness, fear, anger, and disgust), BDI, and State-Trait Inventory	No effect on anxiety, depression (BDI), alertness, and recall of emotional words. HDNI treatment decreased recognition of faces showing disgust and increased recognition of happy faces, and increased recognition of and vigilance to positive words. HDNI increased recognition memory of positive words only in the SAD group. The findings indicate that HDNI treatment produces a positive bias in emotional recall and recognition.
			The emotional test battery consisted of an emotional categorization task with surprise emotional recall and recognition, a facial expression recognition test, and a dot-probe task of attention with masked and unmasked conditions.	

^a^Activation, anxiety, mood.

^b^Relaxation and sleep.

^c^Personal comfort rating.

^d^Depression.

^e^Ions/s converted to ions/cm^3^ for ionizers used in this laboratory based on Terman et al. [8].

^f^Ion concentration in ion/cm^3^ based upon Iwama et al. [41].

*BDI* beck depression inventory; *CgA-like IR* chromogranin A-like immunoreacitivity; *HDNI* high-density negative ions; *SR* self-rating; *STAI-S* state trait anxiety inventory scale; *SIGH-SAD* structured interview guide for the hamilton depression rating scale, seasonal affective disorder.

For reporting purposes, we have organized our review of studies by outcome, ascending year of publication, and the first author’s last name.

### Activation, anxiety, and mood outcomes

Four studies examined the effects of negative and positive air ions on activation, anxiety, and mood [15,16,25,27]. Silverman and Kornbleuh [27] conducted an experiment to examine the effect of negative and positive air ionization on the human electroencephalogram (blinding not reported). Ten healthy adults and two subjects with chronic stationary neurologic conditions participated in the study. Findings indicated a consistent decrease in alpha activity, a non-specific response, ranging from 0.5 to 1.5 cycle decrements during negative or positive air ionization, or both, in 10 subjects (9 healthy; 1 neurologically impaired).

Charry and Hawkinshire [15] examined the effect of positive air ions on mood in 85 subjects (age range: 18–60; mean age: 30) in contrast to ambient conditions in a single-blind experiment and found significantly greater tension and irritability in subjects’ mood states. In particular, ‘ion-sensitive’ subjects showed that activation decreased and reaction times increased during exposure to positive air ions while non-sensitive subjects showed increased activation and no effects on reaction time.

Dantzler et al. [25] reported that ratings of mood on three questionnaires by nine subjects with bronchial asthma (age range: 35–64) were unaffected by exposure to negative and positive ions for 6-hour exposure periods in a double-blind crossover study. In contrast, Gianinni et al. [16] used a double-blind crossover design to evaluate the influence of negative and positive air ions in 14 university-affiliated volunteers and found that positive air ionization significantly increased anxiety, excitement, and suspicion. In contrast, negative air ionization significantly lowered subjects’ extent of suspicion and excitement to those levels attained prior to positive air ion exposure.

Fifteen studies on activation, anxiety, and mood examined the effects of negative air ions only [5,9,10,12,14,18,26,28-35]. Tom et al. [34] utilized a double-blind randomized controlled study to determine the impact of negative air ions on mood in 56 adults (age range: 17–61; mean age: 23). No significant differences were observed between experimental and control conditions. On the other hand, Buckalew and Rizzuto [12] conducted a double-blind randomized controlled trial (RCT) and identified a significant improvement in mood attributed to negative air ionization between experimental (n=12 men) and control (n=12 men) groups (age range: 20–30; mean age: 22.8).

Baron et al. [28] examined the effect of negative air ionization on mood, memory, and aggression as mediated by personality type among 71 male undergraduate students in a single-blind experiment. The authors found that exposure to moderate/high concentrations of negative air ions significantly heightened aggression among subjects classified as Type A, but not Type B. In addition, the authors reported that negative air ionization produced positive shifts in mood when not provoked by an accomplice, but negative shifts in mood when incited.

Deleanu and Stamatiu [29] conducted an experiment of 112 patients with mental disorders (blinding not reported). The overall study goal was to mitigate patients’ symptoms by exposing them to negative aeroionotherapy for 10 to 30 days. The findings suggested that in the majority of treated patients, attenuation or the complete disappearance of anxiety and depressive reactions, including insomnia and general disposition, were identified. In contrast, Hedge and Collis [18] examined the impact of negative air ionization on mood in a double-blind study conducted among 28 healthy women and found no significant benefit of exposure.

Misiaszek et al. [14] explored the influence of negative air ions on eight manic patients (age range: 22–49) in an experimental pilot study conducted in two phases of four subjects each. The first phase was non-blind and the second was double-blind involving collection of data using anxiety and psychiatric metrics. In phase two, three of the four subjects showed score reductions consistent with clinical improvement; however, inference of these findings was impossible due to the limited number of subjects examined. A more recent single-blind experiment by Reilly and Stevenson [33] evaluated anxiety levels among eight healthy men (age range: 19–25) who were exposed to negative air ionization. The results showed no significant effect of air ions on state anxiety pre- or post-exercise [33]. In a single-blind study conducted by Watanabe et al. [35], 13 healthy adults (age range: 21–49; mean age: 26.4) rated their mood after entering a sauna system on two occasions—one with negative air ionization, the other without. The authors observed no significant difference in reported mood states between experimental and control conditions.

Nakane et al. [10] conducted a crossover study (blinding not reported) among 12 female undergraduates (age range: 18–22) to examine the effect of negative air ionization on anxiety and salivary chromogranin A-like immunoreactivity (CgA-like IR), a protein indicator of sympathetic nerve activity. The findings showed that exposure to negative air ions significantly reduced anxiety compared to the positive control while performing a computer-oriented task, but negative air ionization in the post-task period was associated with a non-significant reduction. Similar results were reported for CgA-like IR.

Goel and Etwaroo [5] performed a single-blind RCT to determine the immediate effects of bright light, auditory stimulus, and high-density (n=29) and low-density negative air ionization (n=30) on mood and attentiveness in 118 mildly depressed and non-depressed college students (mean age: 19.4). The results showed that exposure to high-density negative air ionization decreased depressive symptoms, total mood disturbance, or anger within 15 to 30 minutes of exposure; however, low-density exposure did not produce significant effects.

A double-blind crossover experiment by Gianinni et al. [26] exposed 24 manic men (age range: 23–29; mean age: 26.7) to high levels of ambient negative air ions and found a statistically significant reduction in subjects’ manic states. In contrast, Malcolm et al. [32] conducted a single-blind experiment among 30 healthy subjects (age range: 18–28) randomized to receive either high-density negative air ions or a control condition and found no effect of exposure on anxiety. Of note, the clinic that performed the Malcolm et al. [32] study subsequently performed a double-blind RCT of adults (21 patients with SAD and 21 controls) exposed to high-density negative air ions and also reported no effect on measures of visual analogue (mood) or State-Trait Anxiety Inventory ratings [31]. When Malik et al. [9] induced stress in 20 adults (age range: 24–35; mean age: 28.9) in a single-blind study by performing a computer-oriented task, the subjects reported a significant decrease in computer-oriented stress and psychological stress following negative air ionization.

Gianinni et al. [36] researched the effects of positive air ions only in a double-blind crossover study conducted among 12 adult male volunteers and found that anxiety, excitement, and serum serotonin levels significantly increased when exposed.

### Relaxation and sleep

Several studies examined the impact of negative and positive air ionization on relaxation and sleepiness. In the study by Silverman and Kornbleuh [27], more than half of their 12 subjects reported one or more symptoms of dryness of the mouth/upper respiratory tract, relaxation, or sleepiness when exposed to either negative or positive air ionization; however, these responses were more prevalent during negative air ionization. Yaglou [19] conducted a single-blind crossover study in 25 healthy adults (age range: 22–51) and a separate study in 6 arthritic patients (age range: 30–62) to examine the effects of negative and positive air ionization on relaxation. In the first study of 25 adults, 5% reported feeling relaxed when exposed to positive air ions; 17% reported feeling relaxed when exposed to negative air ions; and 21% reported feeling relaxed under control conditions [19]. In the second study, a higher frequency of patients reported feeling relaxed or sleepy, or both, when exposed to negative versus positive air ions [19].

Albrechtsen et al. [37] conducted two single-blind experiments to evaluate the influence of negative and positive air ionization on subjective feelings among two groups: 6 randomly-selected women (age range: 20–30) and 12 adults (age range: 19–45) who appeared to be most sensitive to ionization. Outcomes included subjective assessments on feelings of self-exertion, stuffiness, the unpleasantness of cognitive tasks performed, and sleepiness. Across both studies, no significant effects were identified. Hawkins [38] examined the influence of negative and positive air ionization in an office environment on personal ratings of thermal comfort, stuffiness, alertness, and well-being in a double-blind crossover experiment conducted over 12 weeks. Subjects (n=106) were divided into groups based on areas of variable ionization levels. Hawkins observed that negative air ionization was associated with higher subjective ratings of alertness, atmospheric freshness, environmental/personal warmth, and a reduction in the overall complaint rate by 50%. Positive air ion effects were not explicitly discussed.

Twelve studies examined the association of negative air ions only with relaxation and sleepiness [5,7,8,11-14,22,29,32,34],[39]. Assael et al. [11] conducted a double-blind crossover study to examine the effects of negative air ions on relaxation and alertness among 10 healthy participants (age range: 20–65) and 10 subjects on tranquilizers. The authors found that all patients reported an initial relaxation followed by alertness when exposed to negative air ions.

Three previously mentioned studies on air ions and mood associations also evaluated ion effects on relaxation or sleepiness, or both [12,29,34]. The double-blind experiment conducted by Tom et al. [34] of 56 adults assessed the impact of negative air ions on relaxation (very tense versus very relaxed). Although reported feelings of relaxation were slightly elevated in the experimental compared to the control group, the findings were statistically non-significant. On the other hand, Buckalew and Rizzuto [12] identified a significant increase in relaxation attributed to negative air ionization between experimental and control groups in their double-blind study. In the work of Deleanu and Stamatiu [29], sleep normalization was achieved in 53 of 67 patients with insomnia who were exposed to negative air ions (blinding not reported).

Lips et al. [13] performed a double-blind crossover trial to examine the effect of negative air ions on alertness in 18 healthy adults. Subjects worked in either room one with windows (natural ventilation) or room two with no windows (mechanically ventilated). Lips et al. [13] observed that following exposure to enhanced negative air ions, subjects’ feelings of drowsiness were significantly reduced within both rooms. In the pilot study by Misiaszek et al. [14], all four subjects fell asleep and reported feeling calm following negative air ionization in the first phase of the study (non-blind). In the second phase (double-blind), three of the four subjects fell asleep and one subject appeared less agitated. In both phases, patients’ manic behavior reappeared 5 to 10 minutes post-treatment [14].

Terman et al. [8] conducted a double-blind crossover experiment to examine the effects of timed bright light and negative air ionization on sleep timing in 124 subjects (age range: 18–59; mean age: 39.4), with 20 subjects randomized to high-density and 19 subjects randomized to low-density negative air ionization. The findings showed that exposure to high-density versus low-density negative air ionization did not result in statistically significant differences in sleep patterns. On the other hand, Iwama et al. [39] conducted a double-blind experiment with 44 patients randomized to the control and 51 patients randomized to receive negative air ion treatment (mean age: 40). Five degrees of tension were defined: 1=relaxed; 2=normal tension; 3=mild tension; 4=moderate tension; and 5=severe tension. The authors found that treated patients’ tension reduced significantly and quicker.

Goel et al. [22] conducted a double-blind RCT to evaluate the efficacy of bright light and high-density negative air ionization for non-seasonal chronic depression and sleep in 32 patients (age range: 22–65; mean age: 43.7). The findings showed no significant change in sleep onset between high-density (n=12) and low-density (n=10) negative air ionization; but a significant alteration in sleep offset was noted among the high-density subjects. Similarly, in a single-blind study of light and air ion treatment for depression, Goel and Etwaroo [5] found no significant differences in subjects’ feelings of sedation, pleasantness, or intensity. In a double-blind RCT by Terman and Terman [7], 99 adults with SAD (age range: 19–63; mean age: 40.4) were followed to examine the effects of high- and low-density negative air ionization and light therapy during subjects’ final hours of sleep. Sleep disturbances in 3 of 16 patients in the low-density group were observed, but none in the high-density group.

In a single-blind experiment of 30 healthy subjects (age range: 18–28) randomized either to receive high-density negative air ionization or to a control condition, Malcolm et al. [32] found no effect of air ionization on subjects’ feelings of alertness or calmness. A subsequent double-blind RCT of SAD patients and controls reported no effect on patient alertness and found that negative air ion treatment increased vigilance to unmasked positive items in the visual dot-probe task regardless of patient group [31].

### Personal comfort ratings

Three studies evaluated the impact of negative and positive air ionization on personal comfort [17,37,38]. McGurk [17] examined the effects of negative and positive air ions on self-reported feelings of comfort, ease of working on cognitive tasks, and reactions to the test room environment in 10 college-aged males undergoing a single-blind experimental assessment. All subjects were informed that on some days the air would be ionized; however, subjects remained uninformed about polarity. The findings showed that negative air ion exposure resulted in a notable increase in the proportion of subjects reporting more pleasant feelings, while positive air ion exposure versus the control condition resulted in a significantly higher reporting of unpleasantness.

Findings in the Albrechtsen et al. [37] study found no significant relationship between exposure to high concentrations of negative and positive air ions and feelings of self-exertion, stuffiness, or the unpleasantness of cognitive tasks among 25 healthy subjects or 6 arthritic patients. In contrast, Hawkins [38] observed that negative air ion exposure was associated with higher subjective ratings of alertness, atmospheric freshness, and environmental/personal warmth among office employees working in three different areas of variable air ionization levels (double-blind study).

Several more recent studies [13,35,40] examined the influence of exposure to negative air ions only on personal comfort among adults. Finnegan et al. [40] conducted a single-blind experiment and found no significant effect of negative air ionization on personal comfort among 26 adults working within 5 different rooms of an office building. On the other hand, Lips et al. [13] examined the effects of negative air ion exposure on personal comfort and well-being in a double-blind study of 18 healthy adults who worked in either a room with windows (normal environment) or one mechanically ventilated (ion-depleted environment). The findings showed that following exposure to enhanced negative air ions, subjects’ assessments of both their own well-being and their environments (room pleasantness and comfort) improved significantly at both sites, but failed to result in a significant difference in personal thermal comfort scores. In addition, subjects in the ion-depleted environment failed to experience an improvement in air freshness during negative air ion exposure. In the single-blind, ion-enhanced sauna study by Watanabe et al. [35], no significant differences in the reported feelings of pleasantness between exposure settings were observed.

### Depression

All depression studies evaluated potential alterations only from exposure to negative air ions [4-8,22,24,29,31]. In the study of 112 psychiatric patients by Deleanu and Stamatiu [29], the findings showed that in over 50% of 45 treated patients diagnosed with depression, depressive reactions attenuated or completely disappeared with exposure to negative air ions (blinding not reported). Terman and Terman [6] performed a double-blind RCT among 25 patients (mean age: 38.2) to examine the effects of negative air ions on SAD. Subjects were randomized to low-density (n=13) or high-density (n=12) treatment. The authors found that depression severity decreased (determined using SIGH-SAD) more notably for the high- than the low-density treatment group. Applying a remission criterion of ≥50% reduction in symptom severity, 58% of patients reacted to high-density and 15% reacted to low-density air ion exposure. Terman et al.’s [8] double-blind study of the effects of timed bright light and negative air ionization on SAD in 124 adults showed that exposure to high-density air ionization provided subjects with clinically significant relief by producing a 50% reduction in depressive symptoms from baseline. In addition, the remission rate associated with high-density negative air ionization rose substantially with an additional 10 to 14 days of treatment after the first period, but low-density exposure showed no significant effect [8].

In their double-blind study evaluating the efficacy of bright light and high-density negative air ion exposure for non-seasonal chronic depression in 32 adults, Goel et al. [22] observed a score improvement on the SIGH-SAD of 51% for high-density exposure (remission rate 50%) compared to 17% for low-density exposure (remission rate 0%). Similarly, Goel and Etwaroo’s [5] single-blind study of the immediate effects of bright light (n=29), auditory stimulus (n=30), high-density (n=29), and low-density negative air ionization (n=30) in mildly depressed and non-depressed adults indicated that exposure to high-density negative air ions decreased depressive symptoms within 15 to 30 minutes; however, low-density exposure did not produce any significant effects.

In a double-blind RCT by Terman and Terman [7], 99 adults with SAD or bipolar II disorder were followed to examine the effects of high- and low-density negative air ionization and light therapy during the final hours of sleep. Study findings based on SIGH-SAD indicated that exposure to low-density negative air ions resulted in a significantly lower improvement (22.7%) in depression scores compared to improvement with high-density exposure (47.9%). Flory et al. [4] also investigated the effects of high- and low-density negative air ionization and light therapy on SAD among 73 university-affiliated women (age range: 18–51; mean age: 20.8) in a single-blind RCT and found that subjects in all study groups showed significant score decreases on the SIGH-SAD self-rating scale and the Beck Depression Inventory (BDI) scale. Dauphinais et al. [24] performed a double-blind RCT of adult patients with bipolar depression to examine the effect of negative air ions. Subjects were randomized to low-density (n=20), high density (n=2), or bright light (n=18) treatment for 8 weeks. Of note, the low-density group was considered the control and too few data were available for the high-density group to allow for a meaningful analysis; therefore, data among the high-density group were not reported. The authors found no significant difference between the depression severity scores (determined using SIGH-SAD) of the light and low-density treatment groups (52% vs. 47% reduction, respectively) or between the proportion of responders and remitters (light group—50% of subjects were either responders or remitters; low density ion group—55.6% of subjects in the low-density treatment group were either responders or remitters).

Harmer et al. [31] exposed 21 SAD patients and 21 controls in a double-blind RCT to high levels of negative air ions for 1.5 hours. Post-exposure measures of depression, as measured by the BDI scale, were unaffected by treatment. Additionally, SAD patients, but not controls, exhibited an increased recognition memory for positive words. The overlap in the results of this study with those of Malcolm et al. [32], and parallels between air high-density negative ion treatment and single-dose antidepressant administration on negative affective bias [41,42], suggest a link between emotional processing of certain stimuli and depressive states.

### Meta-analysis of depression studies

**Figure 1:**
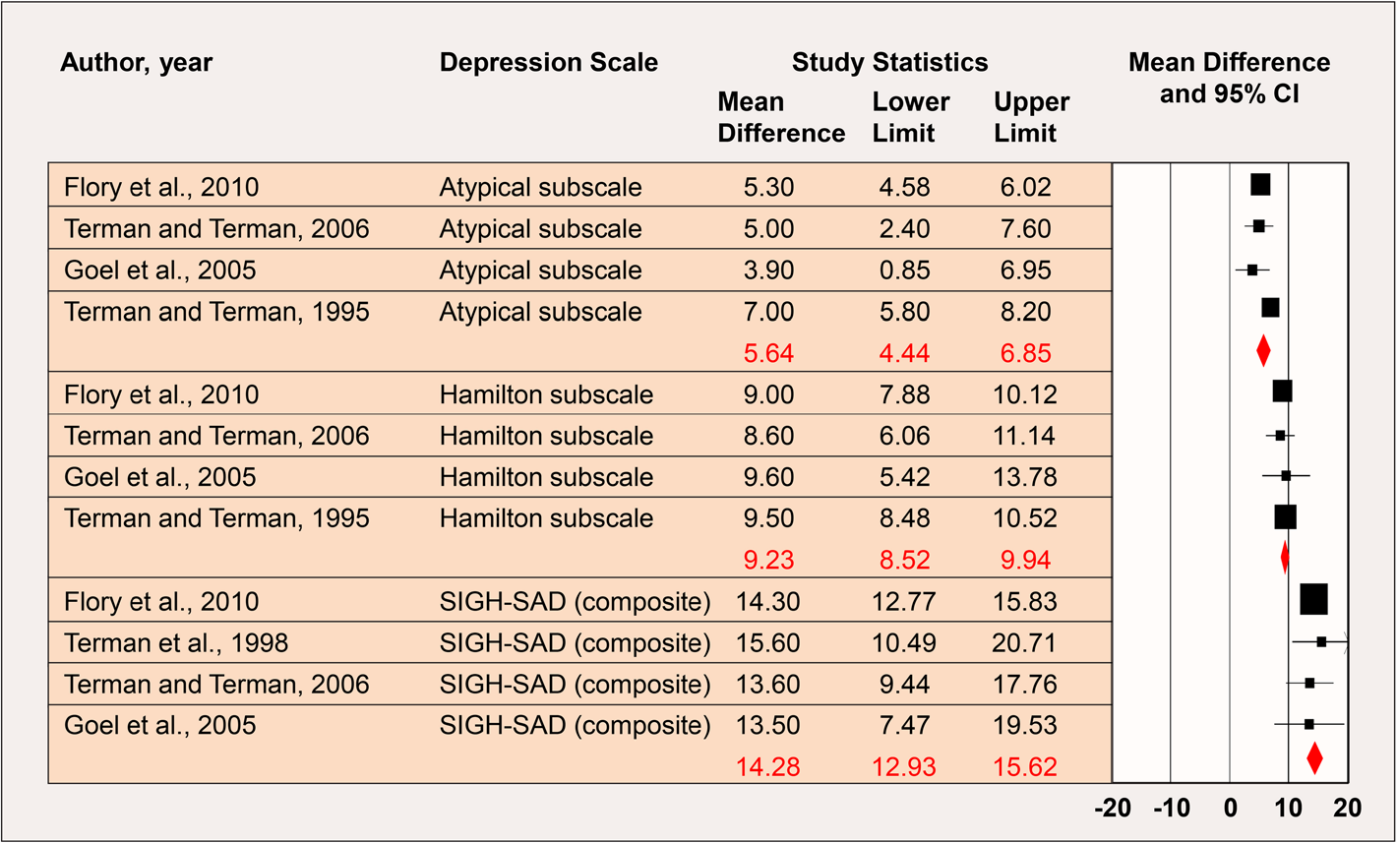
**High-Density Negative Air Ion Exposure and Depression.** *Includes data from studies at the last follow-up time point where applicable [6-8]; *p* for heterogeneity (composite SIGH-SAD) = 0.94. CI: Confidence Interval; SIGH-SAD: Structured Interview Guide for the Hamilton Depression Rating Scale, Seasonal Affective Disorders.

**Figure 2:**
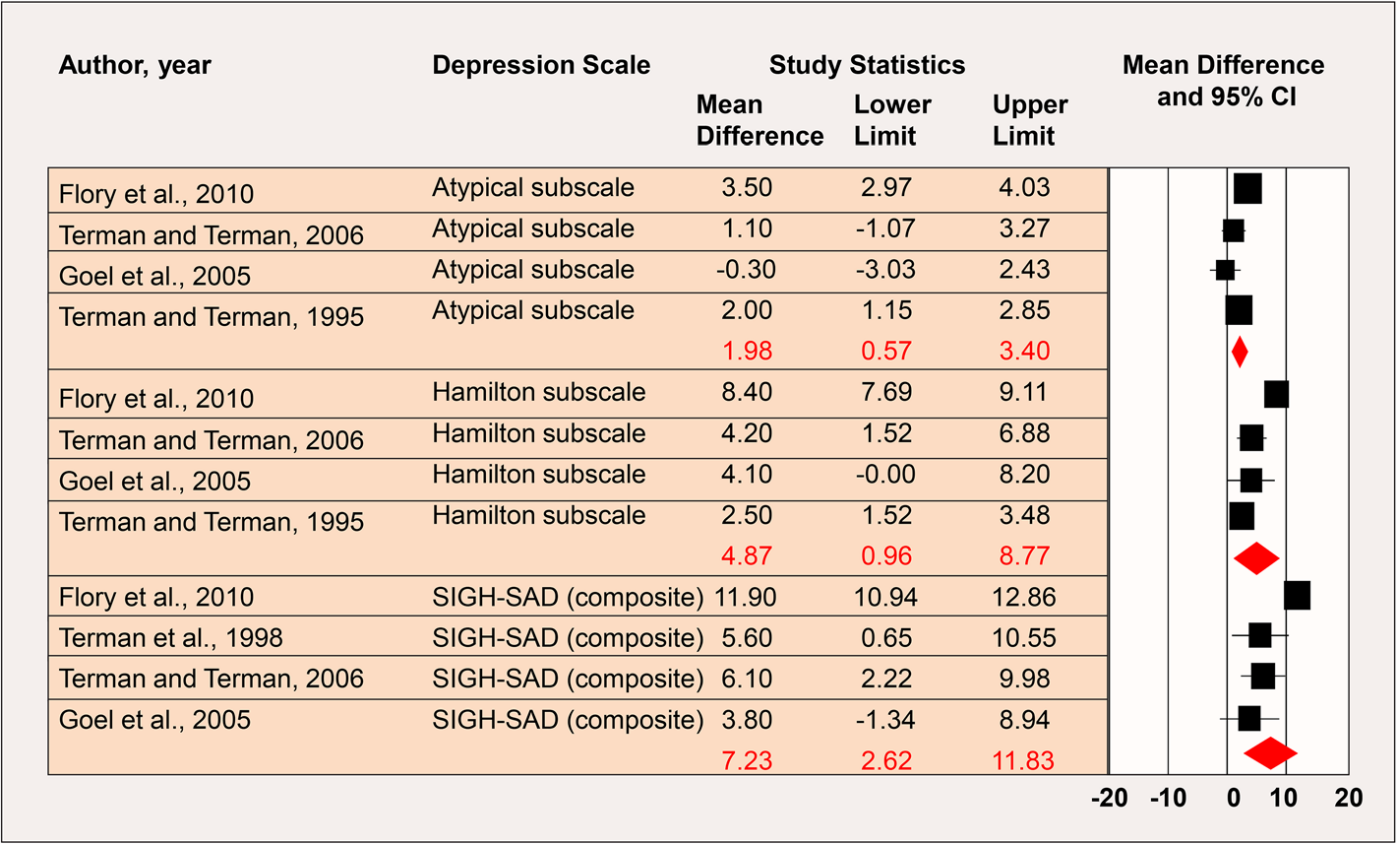
**Low-Density Negative Air Ion Exposure and Depression.** *Includes data from studies at the last follow-up time point where applicable [6-8]; *p* for heterogeneity (composite SIGH-SAD) < 0.0001. CI: Confidence Interval; SIGH-SAD: Structured Interview Guide for the Hamilton Depression Rating Scale, Seasonal Affective Disorders.

The forest plots and overall weighted differences in group means (i.e. *pre-* minus *post-ion exposure* mean scores) by ion concentration (high/low) are shown in Figures 1 and 2. Estimates of treatment effects for studies with multiple follow-up times [6-8] were examined by time point also. Utilizing the later post-baseline mean score where applicable, the weighted differences in group means for the Atypical symptom subscale, Hamilton subscale, and composite SIGH-SAD scale were 5.64 (95% CI: 4.44-6.85), 9.23 (95% CI: 8.52-9.94), and 14.28 (95% CI: 12.93-15.62), respectively (*p* for heterogeneity (SIGH-SAD) = 0.94); thus, the results were indicative of a beneficial effect of high-density negative air ion treatment on SAD and treatment effects were comparable between studies (Figure 1). The weighted differences in group means in the low-density negative air ion analysis for the Atypical symptom subscale, Hamilton subscale, and composite SIGH-SAD scale were 1.98 (95% CI: 0.57-3.40), 4.87 (95% CI: 0.96-8.77), and 7.23 (95% CI: 2.62-11.83), respectively (*p* for heterogeneity (SIGH-SAD) < 0.0001); thus the results were also statistically significant, but smaller in magnitude and were significantly different between studies (Figure 2).

The findings were similar when utilizing the earlier post-baseline mean score reported by Terman and Terman [6,7] and Terman et al. [8] (results not shown); however, the magnitude of effect by subscale and overall was consistently smaller than those shown in Figures 1 and 2. Furthermore, the weighted group mean difference for the Atypical symptom subscale was statistically non-significant in the low-density negative ionization analysis (mean=1.54 (95% CI: -0.31-3.39)).

**Figure 3:**
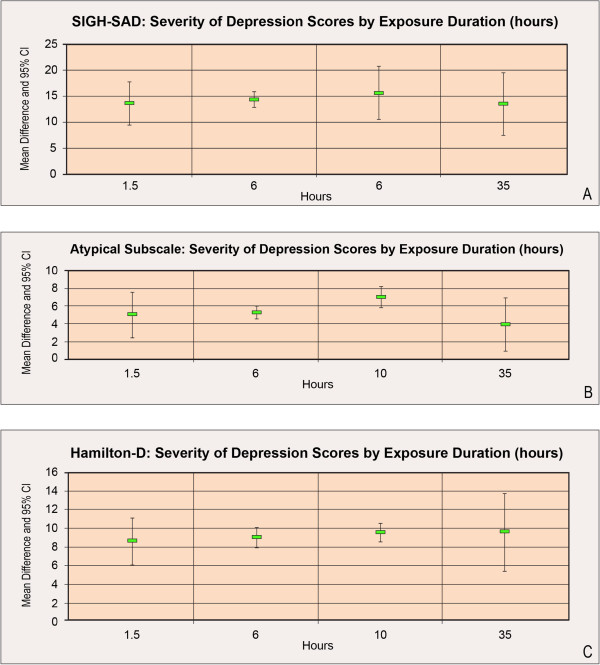
**Dose–response Assessment Between Exposure Duration as Measured by Hours, within High-Density Air Ion Levels, and Each Study’s Score Mean Difference.** *Terman, 1998 [8] only provided data for the composite SIGH-SAD scale and not by subscale; Terman and Terman [6] only provided data by subscale and not for the composite SIGH-SAD scale.

Sensitivity analyses were performed by removing the Terman and Terman [6] study since the data were presented in a figure and not explicitly reported. These analyses showed no alteration in the findings. An additional assessment of exposure duration (hours), within high- and low-density air ion levels, and each study’s score mean difference indicated no evidence of a dose–response relationship (Figure 3).

**Figure 4:**
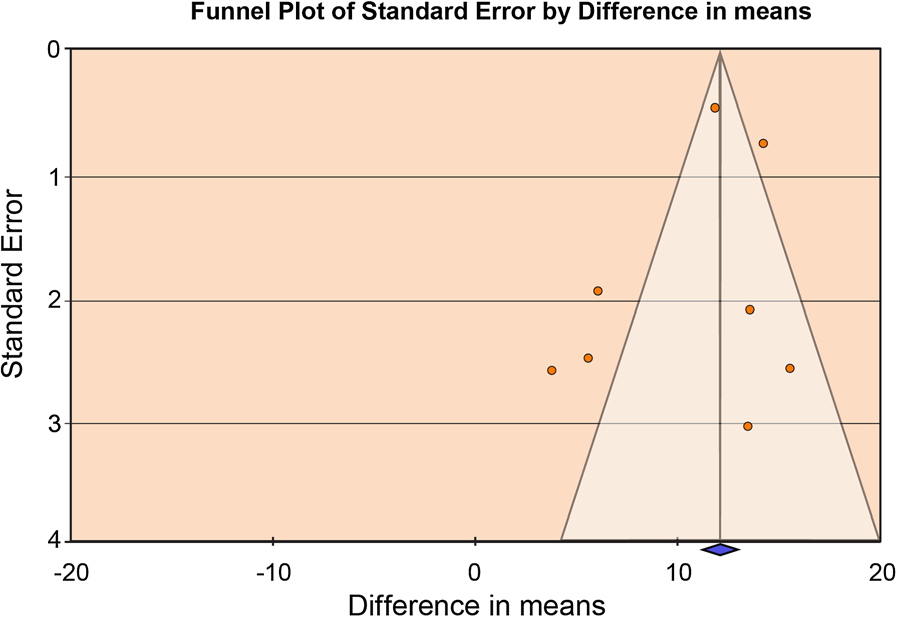
**Visual Assessment of Publication Bias Using a Funnel Plot (SIGH-SAD composite scores).** *A clustering indicative of publication bias around the mean treatment effect was not observed (i.e., no marked asymmetry was evident). In the absence of publication bias we would expect the studies to be distributed symmetrically about the combined treatment effect.

Publication bias was examined visually with funnel plots, which allow for a visual assessment of the estimated intervention effects from the individual studies plotted against a measure of treatment effect size. Separate plots were done for SIGH-SAD composite scores and SIGH-SAD subscales combined since Terman and Terman [6] reported estimates by subscale only and Terman et al. [8] reported estimates for the composite scale only. A clustering indicative of publication bias was not observed (Figure 4) (i.e., no marked asymmetry was evident). Statistical evidence of publication bias was not found (Begg rank correlation *p*=0.71; Egger regression *p*=0.37). These findings were supported by those observed when combining the Atypical and Hamilton subscales.

## Discussion

This review and meta-analysis examined the relationship between negative/positive air ion exposure and emotional state in 33 human experimental studies published from 1957 to August, 2012. To our knowledge, this is the first comprehensive review to summarize the literature on air ionization and psychological outcomes. Also, no studies have previously meta-analyzed the influence of high- and low-density negative air ions on subjects’ depression symptom severity. Our main findings were two-fold. First, we failed to identify a consistent beneficial or detrimental effect of negative or positive air ionization on mental well-being based on studies of anxiety, mood, relaxation/sleep, and personal comfort. Second, our meta-analysis of five studies [4,6-8,22] on negative air ionization and depression suggested a decreased severity of symptom scores in subjects with exposures to high air ion levels. Specifically, we observed a decrease in depression scores, thus corresponding to an improvement in subjects’ depressive state, in comparisons of low- to high-density negative air ion exposure (weighted mean score decrease of 7.23 and 14.28, respectively). A causal basis for this finding, however, was not presumed as the durations of exposure and depression scores were not dose-related.

Exposures to air ions in low dose conditions slightly, but significantly, reduced depression scores measured on the SIGH-SAD. Indeed, no study reported that low dose exposure produced a clinically significant reduction in depression (criterion applied by Terman et al. [8]; Flory et al. [4]; Terman and Terman [6]) of greater than 50%. In fact, it appears that low dose ionization may be regarded by these investigators as an inactive exposure condition [22,24].

Although our meta-analysis showed that exposures to high levels of negative ions was associated with a significant improvement in rated depression severity as measured using SIGH-SAD, a primary metric for both seasonal and non-seasonal depression [4,43-46], and a lack of statistical heterogeneity across study results in the high-density analysis was observed, the findings should be cautiously interpreted. First, this body of work typically did not control for or failed to report on environmental factors affecting exposure including the electric field, air flow, humidity, and temperature. It is well known that the spatial distribution and numbers of air ions vary considerably due to differences in these factors [47]. Hence, the findings summarized herein are likely impacted by unmeasured variables within the available studies and the extent of this impact remains unknown. Second, air ion concentrations for high- and low-density were different across studies, ranging from 4.0 × 10^3^ ions/cm^3^ to 1.0 × 10^4^ ions/ cm^3^ for low-density and from 2.7 × 10^6^ ions/cm^3^ to 2.7 × 10^7^ ions/ cm^3^ for high-density (except for Flory et al. [4], who defined high-density as ≥2.0 × 10^6^ ions/cm^3^). Our finding of statistically significant heterogeneity across studies in the low-density analysis is likely impacted by these varying exposure levels, whereas the effect of high-density air ion treatment may occur independently of the range of exposure levels if an effective exposure threshold is exceeded. Given that at most two studies reported the same air ion concentrations for high- and low-density, however, we could not justify performing separate meta-analyses by ion concentration. However, when hours of exposure were considered as a surrogate for dose within the high- and low-density analyses, repeated or longer exposure durations to negative air ions failed to produce a greater effect on depression scores than did shorter durations. Third, all studies included in the meta-analysis except Flory et al. [4] were conducted by a single research group, which provides little independent replication and may explain, in part, the low between-study variance observed in the high-density analysis. Fourth, differential effects, if any, between men and women were not examined. Gender-stratified analyses are important to consider given that the pharmacokinetics, pharmacodynamics, and hormonal effects between men and women differ and likely influence depression severity [48]. Finally, some depression studies [5,24,29,31] included in our narrative review were unable to be meta-analyzed because of the heterogeneous reporting of available data and the use of different metrics for assessing depression (e.g., BDI and subjective assessments on the amelioration of ‘depressive reactions’). Additional experiments are warranted to clearly understand the impact of negative air ionization on depression severity and how findings may be influenced by variable concentration levels and different metrics for symptom measurement. Future studies should aim to determine the efficacy of high-density air ion therapy for treating depression among men and women. Studies should also aim to evaluate the specificity of any response(s) to negative air ions by testing positive air ions as well.

Based on our review, there is no scientific basis for concluding that air ions have a beneficial or adverse effect on measures of anxiety, mood, relaxation/sleep, and personal comfort in the range of exposures reviewed (200–300 ions/cm^3^ (ambient levels) to 10^6^ ions/cm^3^). The quality of many studies, however, is low and there are several important inconsistencies across studies (e.g. differential study settings/populations, follow-up periods, exposure/outcome measurement and assessment, and unmeasured confounders such as temperature). Of particular importance is the heterogeneity observed in the frequency, duration, and intensity of air ionization evaluated. Presumably, the greater the ion concentration, combined with longer exposure durations at greater frequency, the greater the likelihood for air ion exposure to produce a biological response in exposed subjects, if in fact a real association is present. While there is no consistent support in animal studies for effects of negative or positive air ion treatment on central nervous system neurotransmitter systems linked to depression [21,49,50], Dowdall and De Montigny [51] have reported that continuous exposure of rats to negative air ions at a density of 1.5 × 10^6^ ions/cm^3^ for 21 days increases the response of hippocampal pyramidal neurons to iontophoretically applied serotonin as do several antidepressant drugs. Nonetheless, human studies to date on the relationship between exposure duration, within high and low air ion concentrations, and depression symptom severity do not support such a relationship. In addition, variable distances between subjects and the location at which ion generators were situated likely influenced the number of air ions reaching the subjects. Based on the exposure assessment alone, proper comparison across studies is therefore quite difficult due to the varying exposure assessments, differences in air ion systems used, and disparate monitoring of ion levels. A disparity in the measurement and assessment of the outcomes evaluated also renders a comparison across studies difficult. In this regard, instruments other than the SIGH-SAD to measure depression severity (e.g., the BDI [52], the Center for Epidemiological Studies Depression Scale [53], the Zung Self-Rating Depression Scale [54]) might be considered in future studies since different depression scales may vary in sensitivity and specificity for depression severity, may differ in the measurement of different construct(s) based on the inclusion of specific survey items (i.e., items may discriminate between different dimensions of depression), and may be more suited over others in specific target populations (e.g., young adults vs. elderly patients). Furthermore, no study reported responses to air ion therapy by gender. Specific tests for differential responses, however, would have been of interest given that gender specific-differences are reported in the literature for many emotional parameters [55-57].

Though major limitations of the studies reviewed have been discussed, we acknowledge certain strengths. Since all studies were experimental, most, but not all, observations were made within a controlled environment and prospectively. In addition, participants in 2830 studies remained blind to exposure and ion density, thus mitigating potential bias. Blinding of the experimenters was less common (1618 of 2830). Such precautions should be taken in future studies to minimize introducing possible bias by subjects and investigators. In our review, subject expectations in some studies were compared with depression ratings at the study end. Some studies found no association between expectations and the outcome, suggesting minimal bias [5,8], while other more recent studies reported a significant relationship [4,7].

The World Health Organization conducted a community-based study in 14 countries on the prevalence and severity of mood disorders and found that the 1-year prevalence of mood and anxiety disorders in developed nations ranged from 3.1%-5.3% in Japan to 9.6%-18.2% in the US [58]. Kessler et al. [59] used the National Comorbidity Survey Replication to estimate the lifetime prevalence of DSM-IV disorders and reported lifetime prevalence estimates for mood (20.8%) and anxiety disorders (28.8%). An earlier report by Kessler et al. [60] found that lifetime prevalence for clinical depression among US adults was 16.2% and 1-year prevalence was 6.6%. Globally, the burden of mood disorders such as depression is on the rise, with only 30% of cases worldwide receiving appropriate care for depression [61]. Hence, mood and anxiety disorders present a global crisis that heavily burdens society with serious implications for daily quality of living, economic costs, and the need for individually-tailored treatment.

## Conclusions

Our narrative review provides no basis for further investigation of a variety of emotional state indicators and air ionization. Our meta-analysis, however, strengthens the rationale for further study of high dose negative ionization (>2.7 × 10^6^ ions/cm^3^) on depression severity, an effect, if real, that remains to be fundamentally understood. Such studies should apply a double-blind design with rigorous control over air ionization and potential confounding, including placebo effects. In addition, using validated metrics for outcome assessment in large study populations; determining justifiable thresholds to delineate between sham, low, and high air ion concentrations; and implementing an adequate exposure duration and follow-up period are recommended. Given that longer or repeated exposures to negative air ions were not observed to strengthen the response of subjects, additional investigation of the biological plausibility is warranted. The concentrations of air ions expressed as parts per trillion are vanishingly small and well-controlled animal studies do not report changes in catecholamine neurotransmitter levels [50] or the levels and turnover of serotonin in the brain [49] even though opposing effects of longer-term exposure to negative and positive air ions on the responsiveness of hippocampal neurons to serotonin have been reported [51].

## Competing interests

WHB has consulted for AltaLink LLC, and public and private electric utilities in the preparation of environmental impact assessments and assisted scientific organizations, regulatory agencies, and health agencies to keep abreast of current research involving exposures relating to the use and transport of electricity.

## Authors’ information

VP was awarded her Ph.D. in epidemiology from the University of Michigan at Ann Arbor. Her research primarily focused on the impact of psychological stress on the health of young adults in the community setting. DDA was awarded his Ph.D. in epidemiology from the University of Alabama at Birmingham. He has conducted numerous state-of-the-science reviews and weight-of-evidence assessments on complex medical and scientific issues. WHB was awarded his Ph.D. in neuropsychology from the City University of New York for research at The Rockefeller University, where he continued for postdoctoral research in neurochemistry. He has been involved in air ion research since 1982 and served as a science advisor to the Minnesota Environmental Quality Board and the Vermont Department of Public Service on health and safety issues relating to air ions.

## Authors’ contributions

WHB conceptualized the study and led the design, data acquisition, and interpretation. VP, DDA, and WHB collaborated on the data acquisition, analysis, and interpretation. VP drafted the manuscript. VP, DDA, and WHB provided critical revisions of the manuscript for important intellectual content. WHB provided supervision. All authors read and approved the final manuscript.

## Pre-publication history

The pre-publication history for this paper can be accessed here:

http://www.biomedcentral.com/1471-244X/13/29/prepub
